# 

**Published:** 1916-02

**Authors:** 


### Deaths.

Mrs. J. J. Simmons, of Dallas, died December 27th.

Mrs. W. R. Thompson died in Fort Worth December 19th.

Dr. Charles Clifford Barrows, of New York, died January 2d.

Dr. L. H. Harvey, of Thorndale, died at his home January 15th.

Dr. Leonard Jones, of Harvey, died December 12th.

Dr. Trundeau, noted as the originator of the "fresh air" cure for tuberculosis, died recently.

Dr. T. G. McEachern, of Wills Point, died December 11th. He was a practicing physician for forty-two years.

Dr. A. J. Still died at his home in Kemp January 15th. Dr. Still was one of the founders of the town of Kemp, which is now located on the original homestead of Dr. Still.

Dr. J. P. Hendricks, of Huntsville, died recently. The doctor practiced in Huntsville twenty-one years, and was held in the highest esteem by all who knew and loved him.

Through a resolutions committee, the Harris County Medical Society has issued a memorial on the death of James Donald Duckett, 9-year-old son of Dr. and Mrs. J. D. Duckett, who died recently.

Dr. C. A. Smith, of Texarkana, died January 11th. He was the founder of the local Cotton Belt Employes' Hospital at Texarkana. Dr. Smith was a fine man, and will be greatly missed by those who knew and appreciated his fine qualities.

Dr. Edward Thomas died at the home of his son in San Antonio January 14th. Dr. Thomas was a prominent lecturer and educator.

Dr. J. M. McDuff met with an untimely death near Jourdanton January 10th. His body was forwarded to Atlanta, Texas, his former home, for burial.

